# Microvesicles secreted from equine amniotic-derived cells and their potential role in reducing inflammation in endometrial cells in an in-vitro model

**DOI:** 10.1186/s13287-016-0429-6

**Published:** 2016-11-18

**Authors:** Claudia Perrini, Maria Giuseppina Strillacci, Alessandro Bagnato, Paola Esposti, Maria Giovanna Marini, Bruna Corradetti, Davide Bizzaro, Antonella Idda, Sergio Ledda, Emanuele Capra, Flavia Pizzi, Anna Lange-Consiglio, Fausto Cremonesi

**Affiliations:** 1 Large Animal Hospital, Reproduction Unit, Università degli Studi di Milano, Via dell’Università 6, 26900 Lodi, Italy; 2 Department of Veterinary Medicine, Università degli Studi di Milano, Milano, Italy; 3 Department of Life and Environmental Sciences, Università Politecnica delle Marche, Ancona, Italy; 4 Department of Veterinary Medicine, Università degli Studi di Sassari, Sassari, Italy; 5 Institute of Biology and Agricultural Biotechnology-CNR, Milano, Italy

**Keywords:** Microvesicles, Endometrium, Inflammation, LPS, Regenerative medicine

## Abstract

**Background:**

It is known that a paracrine mechanism exists between mesenchymal stem cells and target cells. This process may involve microvesicles (MVs) as an integral component of cell-to-cell communication.

**Methods:**

In this context, this study aims to understand the efficacy of MVs in in-vitro endometrial stressed cells in view of potential healing in in-vivo studies. For this purpose, the presence and type of MVs secreted by amniotic mesenchymal stem cells (AMCs) were investigated and the response of endometrial cells to MVs was studied using a dose-response curve at different concentrations and times. Moreover, the ability of MVs to counteract the in vitro stress in endometrial cells induced by lipopolysaccharide was studied by measuring the rate of apoptosis and cell proliferation, the expression of some pro-inflammatory genes such as tumor necrosis factor-α (*TNF-α)*, interleukin-6 (*IL-6)*, interleukin 1β (*IL-1β),* and metalloproteinases (*MMP*) 1 and 13, and the release of some pro- or anti-inflammatory cytokines.

**Results:**

MVs secreted by the AMCs ranged in size from 100 to 200 nm. The incorporation of MVs was gradual over time and peaked at 72 h. MVs reduced the apoptosis rate, increased cell proliferation values, downregulated pro-inflammatory gene expression, and decreased the secretion of pro-inflammatory cytokines.

**Conclusion:**

Our data suggest that some microRNAs could contribute to counteracting in-vivo inflammation of endometrial tissue.

## Background

The regular uterine environment promotes normal embryo development, but clinical or subclinical disorders could contribute to pregnancy failure. As reviewed by Hurtgen [1], endometritis is an important cause of reduced fertility in mares in which artificial insemination by fresh or frozen semen may induce acute endometrial inflammatory reactions. If these conditions are not promptly resolved, infections become chronic and, in old pregnant mares, often result in higher pregnancy loss. A similar clinical endometritis also occurs in dairy cows following parturition [2, 3]. Furthermore, cytological endometritis emerged as a problem of remarkable importance for dairy cattle reproduction because animals suffering from this disorder present a persistent inflammatory uterine environment even in the absence of clinical symptoms. A reduced conception rate and increased calving-to-conception intervals are consequences of these uterine diseases [4–7].

Successful implantation requires a complex sequence of signaling events that are crucial to the establishment of pregnancy, and a large number of molecular mediators, influenced by the level of ovarian hormones, have been involved in this early embryo-maternal interaction. These mediators include adhesion molecules, cytokines, growth factors, lipids, and others [8, 9]. Koot et al. [10] underlined that infertility could occur after the early phases of implantation as a malfunction of the endometrium-embryo ‘dialogue’. The degree of endometrial production of these mediators could be impaired by persistent endometritis. Indeed, pro-inflammatory factors transcripted in bovine endometrial epithelial cells are elevated in cases of subclinical or clinical endometritis [11]. Repeat-breeding cows (animals that after three or more inseminations do not get pregnant because of fertilization failure or early embryonic death) show abnormalities in the growth factor-cytokine network, specifically in endometrial epidermal growth factor (EGF) concentration [12]. The EGF family acts on the trophectoderm, promoting cell attachment and embryo development [13], and its impairment could explain the pregnancy failure in these animals.

Many therapies have been proposed to treat or prevent mare endometritis. Post-mating endometritis is usually treated with uterine irrigation and ecbolic, while the acute endometritis treatment is performed with systemic or intra-uterine antibiotics. However, these therapies are not always effective for resolving chronic uterine inflammation. The prevention, mainly in cattle, includes nutritional supplements and hygienic conditions during parturition. Commonly, therapies in use include hormonal treatments with GnRH, exogenous gonadotrophins, and prostaglandins [14], or the exploitation of assisted reproductive techniques, such as in vitro embryo production and embryo transfer. However, in case of infertility due to endometrial damage, the embryo-maternal interaction and the restoration of uterine receptivity could be improved by regenerative medicine treatment.

Regenerative medicine has several applications in the treatment of many pathologies in both human and veterinary medicine. Treatments are based on mesenchymal stem cell (MSC) transplantation but, although engraftment of the transplanted MSCs has been documented in some cases [15–17], only a small percentage of the injected MSCs engraft successfully in various disease models [18–21]. In an irradiated murine model, endometrial regeneration by bone marrow-derived MSCs has been studied showing a low number of cells engrafted in the regenerating endometrium [22]. Consistent with these findings, some studies recently showed that the regenerative ability of MSCs could be attributed to the production of molecules and mediators capable of activating the intrinsic repair processes in the damaged tissues. To date, the conditioned medium (CM) obtained from in vitro cultured MSCs has been proven to be sufficient to stimulate the structural and functional regeneration of cardiac [20, 23], renal [19, 24], spinal cord [25], and tendon [26] tissues. These results indicate that the beneficial effects of MSCs can be attributed to the activation of paracrine mechanisms enabling stimulation of endogenous stem cells. These cells are responsible for ﻿the bioactive soluble factors (lipids, growth factors, and cytokines) known to inhibit apoptosis and fibrosis, enhance angiogenesis, stimulate mitosis and/or differentiation of tissue-resident progenitor cells, and modulate the immune response [27]. In addition to soluble factors, recent findings indicate that extracellular vesicles are released from MSCs inside the CM and that these can be involved as important mediators in cell-to-cell communication [28]. Microvesicles (MVs) have been categorized into exosomes (EXs), released from the endosomal compartment, and shedding vesicles (SVs), which bud directly from the cell membrane. MVs contain various active molecules such as lipids, proteins, mRNA, and microRNA (miRNA) [29]. It has been demonstrated that CM and MVs can be used in vitro and in vivo to repair tissue damage, increasing the healing rate [26, 29, 30]. MVs are involved in a dynamic mutual paracrine communication between the embryonic and the maternal environment at the early stage of pre-implantation embryo development [31]. Equine embryos at day 8 are thought to secrete MVs that can modulate the functions of the oviduct epithelium through transfer of early pregnancy factor (HSP10) and miRNAs [32]. On the other hand, MVs can be secreted from the maternal side, and endometrium-derived MV miRNAs are revealed to have potential targets in biological pathways highly relevant for embryo implantation [33]. Uterine miRNAs are suggested to play a potential regulatory role in the development and progression of bovine subclinical endometritis. Indeed, Hailemariam et al. [34] demonstrated that there is an aberrant expression of 23 miRNAs in cows with subclinical endometritis compared with healthy cows. Furthermore, they observed a similar expression of miRNA patterns in cytobrush samples from sick cows and in vitro cultured endometrial cells challenged by lipopolysaccharide (LPS). This suggests that in vitro endometrial cell culture, treated with LPS, could be an excellent model to test potential regenerative medicine treatments for endometritis. In human medicine, the different pattern of miRNAs between women with and without endometriotic disease have been proposed as biomarkers that could underpin the development of a noninvasive diagnostic test for endometriosis [35].

In this context, the aims of this study were to identify the presence and type of MVs secreted by amniotic mesenchymal progenitor cells (AMCs), and to elucidate whether equine endometrial cells could be targeted by MVs in vitro. In addition, we considered whether MVs are able to counteract an in vitro endometrial cell inflammatory process induced by LPS.

## Methods

### Materials

Uteri samples were collected from horses slaughtered in a national slaughterhouse under legal regulation. Chemicals were obtained from Sigma-Aldrich Chemical (Milan, Italy) unless otherwise specified, and tissue culture plastic dishes were purchased from Euroclone (Milan, Italy).

### Study design

Initially, amniotic cells were isolated and cultured to produce MVs that were characterized using a Nanosight instrument (Nanoparticle tracking analysis, NTA; Nano-Sight Ltd., Amesbuty, UK). Endometrial cells were isolated, and specific endometrial genes were identified by qualitative reverse transcription polymerase chain reaction (RT-PCR). Isolated endometrial cells were used as the target for different concentrations of MVs. Furthermore, the effect of MVs on endometrial cells treated by LPS was analyzed by quantitative RT-PCR (qRT-PCR) expression of inflammatory genes, evaluation of the release of different cytokines, and viability cell tests. Finally, the presence of some miRNAs, regulating inflammation, inside the MVs was evaluated.

### Tissue collection

Allanto-amniotic membranes were obtained at term from normal pregnancies in three mares. Samples of allanto-amnion were transported at 4 °C in calcium- and magnesium-free phosphate-buffered saline (PBS; Euroclone, Milan, Italy) supplemented with 4 mg/mL amphotericin (Euroclone), 100 UI/mL penicillin and 100 mg/mL streptomycin (Euroclone), and were processed within 12 h of collection. The amniotic membrane was mechanically separated from the allantois and the isolation of AMCs was performed as previously reported by Lange-Consiglio et al. [36].

Endometrial samples were obtained during the reproductive season from normal-cycling mares at diestrus stage (early-mid luteal phase). Before slaughtering, 5 ml of blood was collected in heparinized tubes from all mares. After centrifugation, plasma was separated, kept refrigerated, and immediately transported to the laboratory for progesterone determination by a quantitative enzyme linked fluorescent assay (ELFA) based on the MiniVidas (Biomerieux, Firenze, Italy) technology. According to the manufacturer, the measurement range of the assay varied from 0.25 to 80 ng/ml with an intra-assay variation of 4.12 % and an inter-assay variability of 6.32 %.

Only uteri belonging to mares with an obvious corpus luteum on the ovary and progesterone levels between 6 and 20 ng/ml, indicative of the early/mid diestral phase of the estrous cycle [37], were used for endometrial fragment collection and ensuing cell culture.

Tissue fragments for RNA isolation were immediately immersed in RNA Later solution, whereas those destined for cell isolation and the expansion procedure were kept at 4 °C in saline solution supplemented with 4 μg/ml amphotericin B, 100 IU/ml penicillin, and 100 μg/ml streptomycin and processed within 8 h.

### Cell isolation

Amniotic membrane-derived mesenchymal cells were isolated as recently reported by Lange-Consiglio et al. [36]. Briefly, amnion fragments were incubated for 9 min at 38.5 °C in PBS containing 2.4 U/mL dispase (Becton Dickinson, Milan, Italy). After a resting period (5–10 min) at room temperature in high-glucose Dulbecco’s modified Eagle’s medium (HG-DMEM; EuroClone, Milan, Italy), supplemented with 10 % heat-inactivated fetal bovine serum (FBS) and 2 mM l-glutamine, the fragments were digested with 0.93 mg/mL collagenase type I and 20 mg/mL DNAse (Roche, Mannheim, Germany) for approximately 3 h at 37 °C. The amnion fragments were then removed, and mobilized cells were passed through a 100-μm cell strainer before being collected by centrifugation at 200 × g for 10 min.

Endometrial cells from diestrum uteri of mares were obtained according to the protocol described by Donofrio et al. [38] and slightly modified for equine cells. Briefly, the endometrium was digested in sterile filtered Hank's buffered salt solution supplemented with 2 mg/ml collagenase II, 4 mg/ml bovine serum albumin, and 0.4 mg/ml DNase I for 90 min at 38.5 °C in a shaking bath. Cells were then filtered through a membrane with a pore size of 80 μm and centrifuged at 200 × g for 10 min, then washed twice in PBS. This protocol allowed for the isolation of the endometrial stromal portion. Before seeding, cells were counted using a Burker chamber with the Trypan Blue dye exclusion assay.

### Cell expansion

Endometrial cell (EDC) cultures were established in HG-DMEM supplemented with 10 % FBS, penicillin (100 UI/ml), streptomycin (100 μg/ml), 0.25 μg/ml amphotericin B, and 2 mM l-glutamine. Medium was supplemented with 10 ng/ml EGF for AMC cultures.

To remove non-adherent cells for both cell lines the medium was replaced for the first time after 72 h, and then changed either twice per week thereafter or according to the experiment requirements. For maintenance of cultures, cells were plated in flasks of 25 cm^2^ at a density of 1 × 10^5^ cells/cm^2^ and incubated at 38.5 °C in a humidified atmosphere with 5 % CO_2_. Adherent cells were detached with 0.05 % trypsin-EDTA just prior to reaching confluence (80 %) and then reseeded for culture maintenance at a density of 1 × 10^4^ cells/cm^2^.

A detailed characterization of these cells was performed in the paper of Corradetti et al. [39]. In this study, a molecular characterization of EDCs was performed only at passage (P)0 as a de facto control for gene expression.

### Isolation and measurements of MVs

MVs were obtained from the culture media of AMCs derived from three different placentas, cultured for 1 week with HG-DMEM supplemented with 10 % MV-deprived FCS and overnight in HG-DMEM deprived of FCS and supplemented with 0.5 % BSA (Sigma). The overnight culture media were pooled and centrifuged at 2000 **×** g for 20 min to remove debris, then at 100,000 **×** g (Beckman Coulter Optima L-100 K ultracentrifuge) for 1 h at 4 °C, washed in serum-free medium 199 containing N-2-hydroxyethylpiperazine-N-2-ethanesulfonic acid (HEPES; 25 mM) and submitted to a second ultracentrifugation under the same conditions. After ultracentrifugation, the pellet was immediately resuspended in HG-DMEM, and a fraction of the resuspended pellet was taken for measurements of MV size and concentration. A second fraction was labeled with fluorochrome PKH-26 and the remaining part of the pellet was cryopreserved with 1 % dimethylsulfoxide at –80 °C and used for the in vitro test. The size and concentration of MVs were evaluated by the Nanosight LM10 instrument, which permits discrimination of microparticles less than 1 μm in diameter. The software (NTA 2.0 analytic software) allows the analysis of video images of particle movement under Brownian motion and the calculation of diffusion coefficient, sphere equivalent, and hydrodynamic radius of particles by using the Strokes–Einstein equation. This instrument was configured with a 405-nm laser and a high-sensitivity sCMOS camera (OrcaFlash2.8, Hamamatsu C11440, NanoSight Ltd). Videos were collected and analyzed using the NTA software with the minimal expected particle size, minimum track length, and blur setting all set to automatic. Ambient temperature was recorded manually and did not exceed 25 °C. Each sample (5 μl) was diluted in sterile physiological solution to a final volume of 1 ml. Samples were analyzed within 15 min of the initial dilution with a delay of 10 s between sample introduction and the start of the measurement. For each sample, multiple videos of 30 s duration were recorded generating replicate histograms that were averaged.

### MVs labeling with PKH-26

To trace in vitro MVs by fluorescence microscopy, MVs from AMCs were labeled with the red fluorescence aliphatic chromophore intercalating into lipid bilayers PKH26 dye (Sigma). Briefly, after ultra-centrifugation, the MV pellet was diluted to 1 ml with PKH-26 kit and 2 μl of fluorochrome was added to this suspension and incubated for 30 min at 38.5 °C. At the end of the reaction, 7 ml of serum-free DMEM was added to the suspension that was ultra-centrifuged again at 100,000 g for 1 h at 4 °C. The final pellet was immediately resuspended in HG-DMEM.

### Incorporation of MVs in endometrial cells

To study the incorporation capacity of MVs into endometrial cells, a dose-response growth was performed in three replicates. Endometrial cells were seeded at a density of 60 **×** 10^3^ on culture slides (13 mm; Nalgen Nunc International, Rochester, NY, USA) in 24 wells and co-cultured with 10, 20, 30, 40, and 50 **×** 10^6^ MVs/ml labeled with PKH-26 dye, and pre-incubated or not with trypsin (0.5 mM) for 24, 48, and 72 h at 38.5 °C. At the end of each experimental condition, cells were nuclear stained with 10 μg/ml Hoechst 33343 for 15 min at 38 °C. The uptake of MVs was evaluated by an Olympus BX51 microscope equipped with a Scion Corporation 1394 video camera interfaced with a computer provided with software for image acquisition and analysis (Image-Pro Plus 5.1-Media Cybernetics, Immagini & Computer, Bareggio, Italy). Excitation wavelength was positioned at 550 nm while emission wavelength was set at 567 nm. Hoechst 33342 dye (Sigma) was excited at 353–365 nm while the emission wavelength was set at 460 nm. To detect the intensity of fluorescence, a semi-quantitative analysis was performed. Different images were acquired for each condition and then, for each image, the area of interest (AOI; where the signal was present) was manually defined by the user. Inside the AOI, up to three different background signals were sampled. The background areas were positioned by the user only where the fluorescent signal was not specific. The maximum value collected from the background areas was then used to define the threshold. Only fluorescence with an intensity above the threshold was considered to indicate fluorescence due to labeled MVs. Finally, the program measured the signal intensity expressed in arbitrary units (a.u.).

Confocal microscopy analysis to assess internalization of MVs was performed using a Leica SP2 laser scanning confocal microscope (Leica Microsystems Srl, Italy) equipped with a PL Fluotar 20× AN 0.5 Dry objective.

### In vitro effect of MVs on endometrial cells treated with LPS

The dose-response curve of LPS on endometrial cells was studied showing that 10 ng/ml and 12–24 h were the dose and the times most effective in inducing cellular stress evaluated by an apoptotic study (data not shown). Sixty thousand cells were incubated at the same time with LPS 10 ng/ml and 40 × 10^6^ MVs/ml for 3, 12, and 24 h. In another experimental condition, endometrial cells were treated first for 3 h with LPS and then with MVs at the same concentrations and times. In the last experiment, endometrial cells were treated first for 24 h with MVs and then with LPS 10 ng/ml. Endometrial cells alone or endometrial cells with LPS or MVs only were used as controls at different times. At the end of each experimental condition, the MTT reduction assay method and apoptotic test were used to analyze cell proliferation and viability of cells on some samples. Cells from other samples were detached with 0.05 % trypsin-EDTA, centrifuged, and cryopreserved for molecular biology studies in liquid nitrogen using standard cryopreservation protocols. The supernatants were destined for the evaluation of cytokines released from endometrial cells. All experiments were performed in three replicates.

### Viability cell tests

#### Cell proliferation test by MTT reduction assay method

The MTT reduction assay method (Chemicon, Temecula, CA, USA) estimates the activity of the enzyme dehydrogenase by converting the MTT compound (3-(4,5-dimethylthiazol- 2-yl)-2,5-diphenyletrazolium bromide) into formazan by the mitochondria. The measurement was performed with a spectrophotometer (Perking Elmer HTS 700 plus; Boston, MA, USA) at the absorbance reading of 570 nm for each sample. Briefly, at each experimental condition of in vitro effect of MVs on endometrial cells treated with LPS, cells were washed twice in PBS, and 1 ml of 5 mg/l MTT solution was added to each well. Avoiding light, plates were then placed in a humidified incubator at 37 °C for 4 h. The supernatant was discarded, 1 ml of dimethylsulfoxide was added as an extracting solution, and plates were incubated for 2 h until the precipitations were resolved completely for spectrophotometric reading. This test was performed in three replicates.

#### Apoptotic test

The percentage of apoptotic cells was assessed using an Annexin-V-FITC Apoptosis Detection KIT (Sigma) following the manufacturers’ instructions; 500 μl of cells (5 **×** 10^5^ cells) were incubated with 5 μl of Annexin V solution and with 10 μl propidium iodide for 1 h at room temperature while protected from light. Apoptosis rates were evaluated by conventional fluorescence analysis using a BX 51 microscope (Olympus) equipped with a DMU filter set. One hundred cells were analyzed using a combination of 488/560 nm emission. Cells at the early stage of apoptosis stained with the annexin V-FITC alone. Live cells showed no staining with either propidium iodide or Annexin V-FITC. Cells dead for apoptosis were stained by both propidium iodide and Annexin V-FITC, and cells dead for necrosis were stained by propidium iodide alone. The apoptotic test was performed in three replicates.

### Molecular biology studies

#### Characterization of endometrial cells

After isolation from endometrial tissue, cells were analyzed to detect the expression of specific endometrial genes as previously reported [39]. Total RNA was extracted from endometrial cells immediately after isolation (P0) using TRI Reagent Solution (Life Technologies, Monza, Italy) and conventional RT-PCR was performed with RBC Taq DNA Polymerase (RBC Bioscience) using previously optimized primers [39]. The primer sequences and conditions are shown in Tables 1 and 2. Glyceraldehyde-3-phosphate dehydrogenase gene (*GAPDH*) was employed as a reference gene.

**Table 1:** Oligonucleotide sequences used for reverse transcription polymerase chain reaction analysis

Reference sequence	Markers	Forward	Reverse	Annealing temperature	bp
XM_001498494.3	Progesteron Receptor (*PR*)	GTCAGTGGACAGATGCTGTA	CGCCTTGATGAGCTCTCTAA	55 °C	255
NM_001256979.1	Membrane-associated progesterone receptor (*MPR*)	GCCAAGTATCGTTACCGGAG	AAGAGGATCTGGAGCGTGTG	55 °C	173
XM_001914705.2	Progester one receptor membrane component 1 (*PGRMC1*)	TCAACGGCAAGGTGTTCGAC	GGCTCTTCCTCATCTGAGTA	58 °C	280
XM_003364827.1	Homeobox protein Hox-A9-like (*Hoxa9*)	ACGCTGGAACTGGAGAAAGA	CTTTCGCTCGGTCCTTATTG	55 °C	160
NM_001163856.1	Glyceraldehyde-3-phosphate dehydrogenase (*GAPDH*)	AGATCAAGAAGGTGGTGAAG	TTGTCATACCAGGAAATGAGC	59 °C	178
NM_001081847.2	Matrix metallopeptidase 1 (*MMP-1*)	ACTGCCAAATGGACTTCAAGCTGC	TCTTCACAGTGCTAGGAAAGCCG	60 °C	158
NM_001081804.1	Matrix metallopeptidase 13 (*MMP-13*)	CTCTGGTCTGCTGGCTCACGC	CCAAACTCGTGTGCAGCGAC	60 °C	132

**Table 2:** Oligonucleotide sequences synthesized by Life Technologies

Assay ID	Gene symbol	Entrez gene ID	NCBI assembly	CHR	Location on NCBI genome assembly	Location on transcript or gene
Ec03467871_m1	*TNF*	100033834	2.1	20	31359792	318
Ec03468680_m1	*IL-6*	100034196	2.1	4	54424314	468
Ec04260298_s1	*IL-1B*	100034237	2.1	Un|NW_001871813.1	5795	254

#### Gene expression of pro-inflammatory cytokines

Genes involved in the inflammatory process, such as interleukin-1β (*IL-1β*), interleukin-6 (*IL-6*) and tumor necrosis factor α *(TNF-α*), were analyzed by qRT-PCR under all experimental conditions. The mRNA expression levels of all genes were measured in three samples (biological replicates). Total RNA was isolated using the mirVana™ miRNA isolation Kit (Life Technologies) according to the manufacturer’s protocol and stored at −20 °C. The concentration and purity of RNAs were evaluated three times by the NanoQuant spectrophotometer (Thermo Scientific, USA) and, in order to verify the integrity of extracted RNA, eight samples that were randomly chosen were analyzed on a Bioanalyzer 2100 using the Agilent RNA 6000 Pico Kit (Agilent). According to the RNA quantity, each sample was normalized to the final RNA concentration of 10 ng/μl.

RT-PCRs were performed with the High Capacity cDNA Reverse Transcription Kit (Applied Biosystems/Life Technologies, Carlsbad, CA, USA) using 100 ng of RNA per reaction.

All the qPCR experiments were run in triplicates (technical replicates) using the qPCR protocol described by TaqMan Fast Gene Expression Assays (Life Technologies™) on a 7500 Fast Real-time PCR System instrument (Applied Biosystems by Life Technologies™). To assess gene expression, each target gene and the *GAPDH*, as the housekeeping control gene, were co-amplified. The assay primers were available and synthesized by Life Technologies™.

Average target gene threshold cycle (ΔCt_g_) for each sample (calculated using the CT values of the technical replicates within each experimental conditions) were normalized to the average GAPDH values (ΔCt_GAPDH_) of the same cDNA sample. Then the expression variations calculated were normalized to the internal control (i.e., control cell at 3 h) using the ΔΔCt method. Finally, the fold-change expression of each gene was calculated as 2^−ΔΔCT^ [40].

#### Gene expression of metalloproteinases

Matrix metalloproteinase 1 (*MMP-1*) and matrix metalloproteinase 13 (*MMP-13)* were selected to evaluate the effect of MVs to contrast LPS activity. Gene expression was performed with the SYBR green method in a MyiQ iCycler thermal cycler (Biorad). Triplicate PCR reactions were carried out for each sample, analyzed using primer sequences reported in Table 1. The reactions were set on a strip in a final volume of 25 μl by mixing, for each sample, 1 μl of cDNA, 12.5 μl of 2× concentrated SYBR Premix Ex Taq II (Takara Bio) containing SYBR Green as a fluorescent intercalating agent, 0.2 μM forward primer, 0.2 μM of reverse primer, and MQ water. PCR efficiencies were tested and found to be close to 1. The thermal profile for all reactions was 30 s at 95 °C and then 40 cycles of 5 s at 95 °C, and 30 s at 60 °C. Fluorescence monitoring occurred at the end of each cycle. The efficiency of amplification for each primer was monitored through the analysis of serial dilution. Additional dissociation curve analysis was performed, and in all cases showed a single peak. The data thus obtained were analyzed using the iQ5 optical system software version 2.0 (BioRad). The expression of each gene was normalized to the reference gene *GAPDH* in order to standardize the results by eliminating variation in cDNA quantity. Sequences used are listed in Table 1.

### miRNA analyses by RNA extraction and PCR amplification

The MV pellet was subjected to RNase digestion to remove extraneous ribonucleic acids [41]. Total RNA was isolated from a pool of different MVs and amniotic-derived cell preparations using the NucleoSpin® mRNA kit (Macherey-Nagel, Germany), in combination with TRIzol® lysis and purification of small and large RNA in one fraction (total RNA). RNAs were quantified using a NanoDrop ND-1000 spectrophotometer (NanoDrop Technologies, Wilmington, DE, USA). RNA quality was checked using the Agilent Bioanalyser 2100 (Agilent, Santa Clara, CA, USA), where the presence of small RNAs was verified in both MV and cell samples.

RNAs from all samples were reverse transcribed with the miScript Reverse Transcription Kit and the cDNA was then pre-amplified using the miScript PreAMP PCR Kit (all from Qiagen, Valencia, CA, USA), following the manufacturer’s instruction with some modification: miScript PreAMP Primer Mix was replaced with miR-specific primers: hsa-miR-26a-2, -335, -146a, and SNORD95 as forward primer, and miScript Universal Primer as reverse primer in separate reactions. *Homo sapiens* hsa miRNA were perfectly homologous with *Equus caballus* eca miRNA sequence. PCR was performed on pre-amplified products using the PCR Master Mix (2×) (Thermo Fisher Scientific Inc., Waltham, MA, USA), with the same primer couple: hsa-miR-26a-2, -335, -146a, SNORD95 in combination with miScript Universal Primer. The small nucleolar snoRNA, C/D Box 95 SNORD95 was used as the positive control. Negative controls using water in place of the pre-Amp product were performed alongside each reaction. The cycling conditions were 3 min at 95 °C, followed by 35 cycles of 30 s at 95 °C, 30 s at 58 °C, 1 min at 72 °C, and finally 7 min at 72 °C. The amplified PCR products were separated electrophoretically on 2.5 % agarose gels, and visualized under UV, using the GeneRuler 50 bp as a DNA ladder (Thermo Fisher Scientific Inc.).

### Cytokines

Cytokine release (IL-6, transforming growth factor (TGF)-β, and TNF-α) was measured in cell-free supernatants obtained by centrifugation at 1200 rpm for 5 min and stored at −80 °C until measurement. Cytokine production was assessed by commercially available sandwich ELISAs (Bioptis SA, Liege, Belgium). ELISAs were performed according to the supplier’s instructions. Results are expressed in pg/ml. The limit of detection was 15.6 pg/ml for all cytokines tested.

### Statistical analysis

For quantitative PCR experiments, data were analyzed by one-way analysis of variance (ANOVA). Also, cell viability data were analyzed by one-way ANOVA applying a Bonferroni correction.

For cytokines, statistical differences were determined using ANOVA followed by Dunnett’s multiple comparison test, the Tukey–Kramer multiple comparisons test or unpaired *t* test.

Differences were considered statistically significant if the value of *P* was <0.05.

## Results

### Tissue collection and cell isolation

**Fig. 1:**
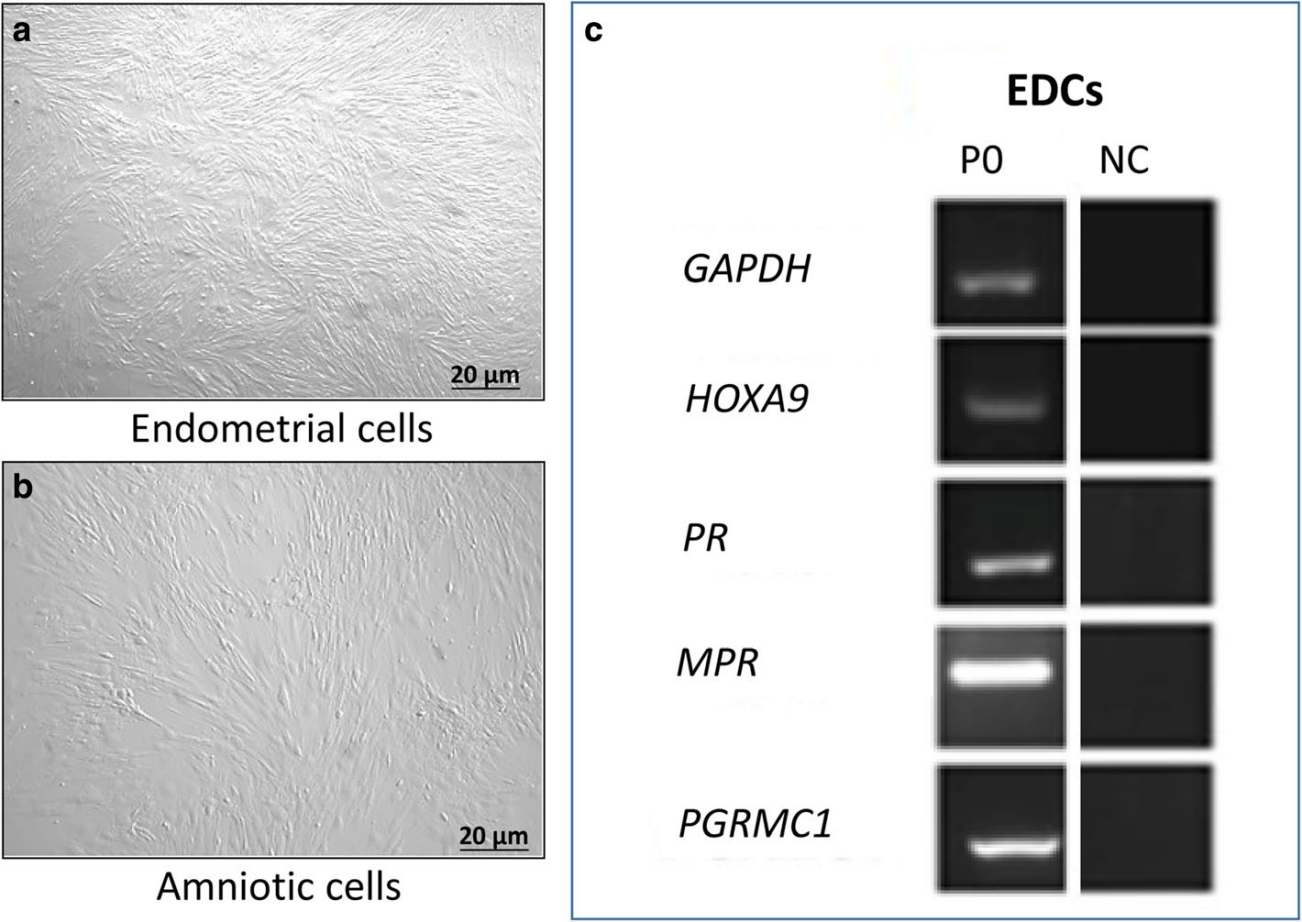
Morphology of **a** endometrial (*EDCs*) and **b** amniotic cells. **c** Expression of specific endometrial markers revealed by qualitative PCR on endometrial cells at passage 0 (*P0*). *GAPDH* glyceraldehyde-3-phosphate dehydrogenase, *HOXA9* homeobox protein Hox-A9-like, *MPR* membrane-associated progesterone receptor, *NC* negative control, *PGRMC1* progesterone receptor membrane component 1, *PR* progesterone receptor

Cells were selected for their ability to adhere to plastic. For AMCs, the initial viability was >90 %, whereas for EDCs it was >85 %. EDCs (Fig. 1a) and AMCs (Fig. 1b) displayed fibroblast-like morphology. Molecular biology analyses at P3 showed that AMCs showed a typical mesenchymal stromal phenotype, with the expression of markers such as CD29, CD44, CD106, CD105, and MHCI, but not CD34 and MHCII. Moreover, AMCs showed differentiative potential in mesenchymal (osteogenic, adipogenic, and chondrogenic) and ectodermic lines (neurogenic) as reported by Lange-Consiglio et al. [36].

The molecular biology study on endometrial cells at P0 confirmed that these cells were endometrial cells because of the expression of *PR, MPR, PGRMC1, HOXA-9* (Fig. 1c).

### Isolation and measurement of MVs

In all the studied samples, the viability of AMCs at the time of MV collection was 99 % as detected by trypan blue exclusion. By Nanosight, the size of MVs ranged from 50 to 670 nm, with a mean size of 258 ± 55 nm for three samples. The number of MVs ranged from 800 to 4700 particles/cell, with a mean value of 2550 ± 71 particles/cell (corresponding to 540 × 10^6^ particles/ml of medium). In a previous study [42], transmission electron microscopy (TEM) analysis revealed the presence of variably sized extracellular membranous vesicles budding from, or lying near, the cell of origin. The size of MVs ranged from 100 nm to 1000 nm, with a predominance of vesicles between 100 and 200 nm. Because of the size, by Nanosight and TEM, and morphological characteristics, the vesicles observed were mainly considered as shedding vesicles.

### Incorporation of MVs in endometrial cells

**Fig. 2:**
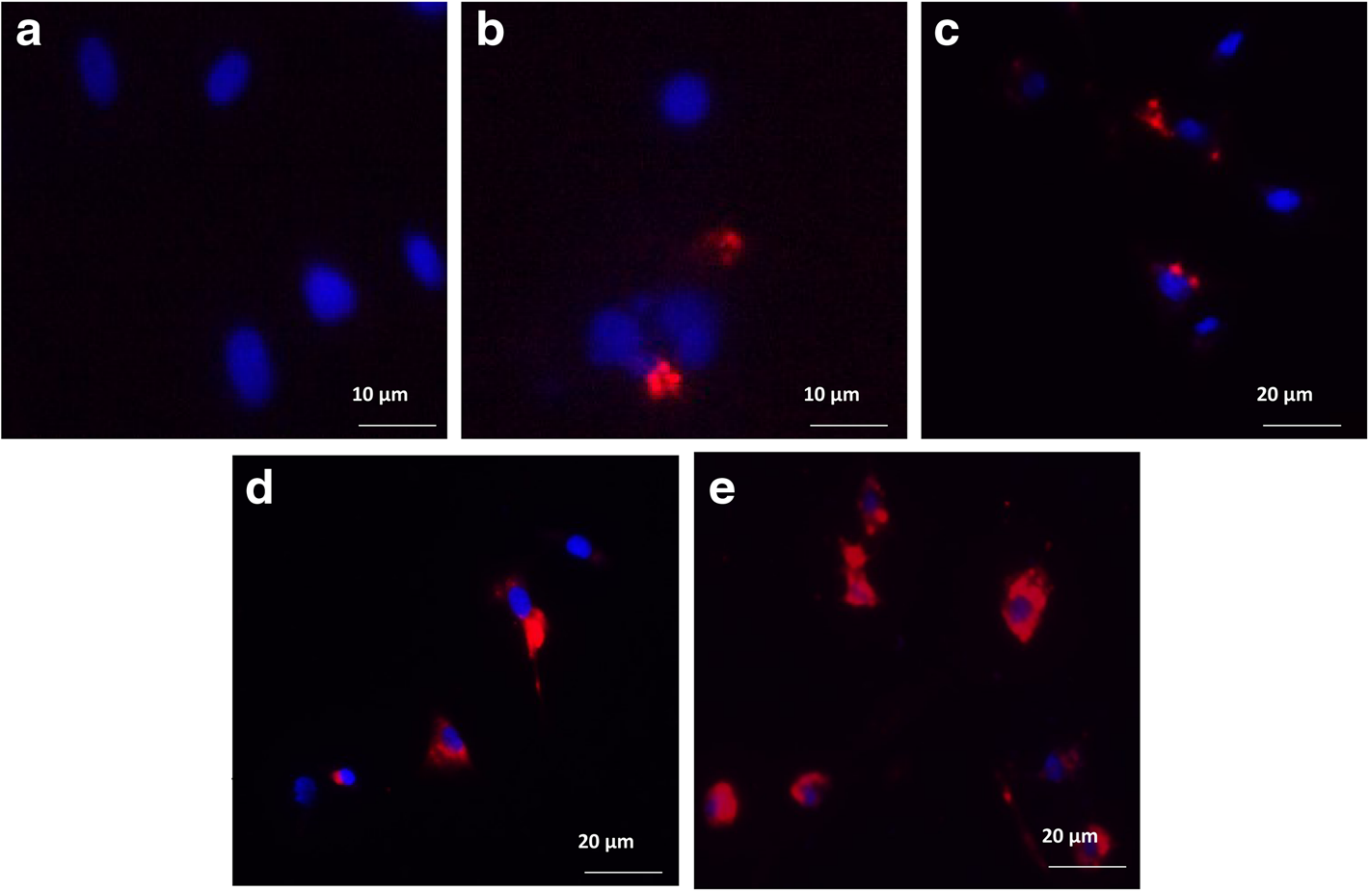
Representative micrographs of internalization by endometrial cells of MVs labeled with PKH-26. Under a fluorescent microscope, the endometrial cell nuclei are *blue* and the MVs are *red*. **a** No incorporation of MVs pre-incubated with trypsin. Incorporation of 40 **×** 10^6^ MVs/ml into the target cells from **b** 24 h, **c** 36 h, **d** 48 h, and **e** 72 h, respectively

**Fig. 3:**
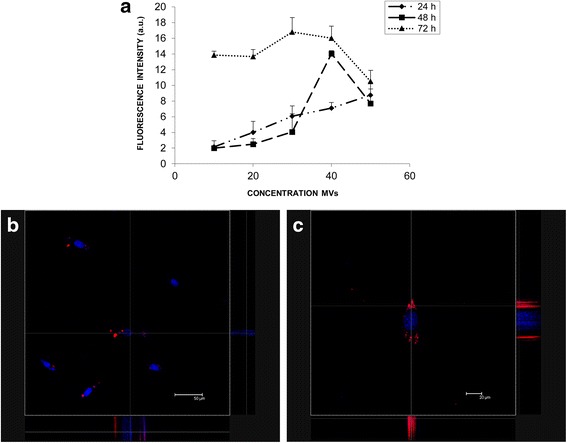
MV uptake. **a** Graph of dose/exposure time for microvesicle (*MV*) incorporation. The uptake of MVs drastically increased at 72 h for all concentration but decreased at 50 × 10^6^/ml MVs. Data represent the mean and SD of three independent experiments. **b**, **c** Representative z-stack orthogonal projection micrographs showing the internalization of MVs as detected by confocal microscopy in two group of endometrial cells co-cultured with MVs for 24 h. The images were taken at different plans scanned every 0.5 μm from top to bottom of the nucleus and showed at different magnification. *a.u.* arbitratry units

As seen by fluorescence microscopy, in all the studied samples no fluorescence signal was detectable up to the sixth hour of co-incubation of MVs with endometrial cells, and only nuclei stained with Hoechst 33342 were visible (Fig. 2a). The increase in uptake of 40 × 10^6^ MVs/ml by endometrial cells between 24 h and 72 h is showed in Fig. 2b, c, d and e. No signal was detected after treatment of MVs with trypsin. The incorporation of MVs is gradual and constant at 24 h with a concentration of 40 × 10^6^ MVs/ml, and suddenly increases at a concentration of 50 × 10^6^ MVs/ml. The uptake of MVs drastically increased at 48 h at a concentration of 40 × 10^6^ MVs/ml and decreased at a concentration of 50 × 10^6^ MVs/ml. The internalization and accumulation of MVs peaked at 72 h for all the different concentrations but, once again, decreased at a concentration of 50 × 10^6^ MVs/ml (Fig. 3a).

As seen by confocal microscopy, after 24 h of incubation with MVs, endometrial cells showed a fine granular fluorescent pattern within their cytoplasm, indicating incorporation of MVs (Fig. 3b and c).

#### In vitro effect of MVs on endometrial cells treated with LPS

##### Viability cell tests

**Fig. 4:**
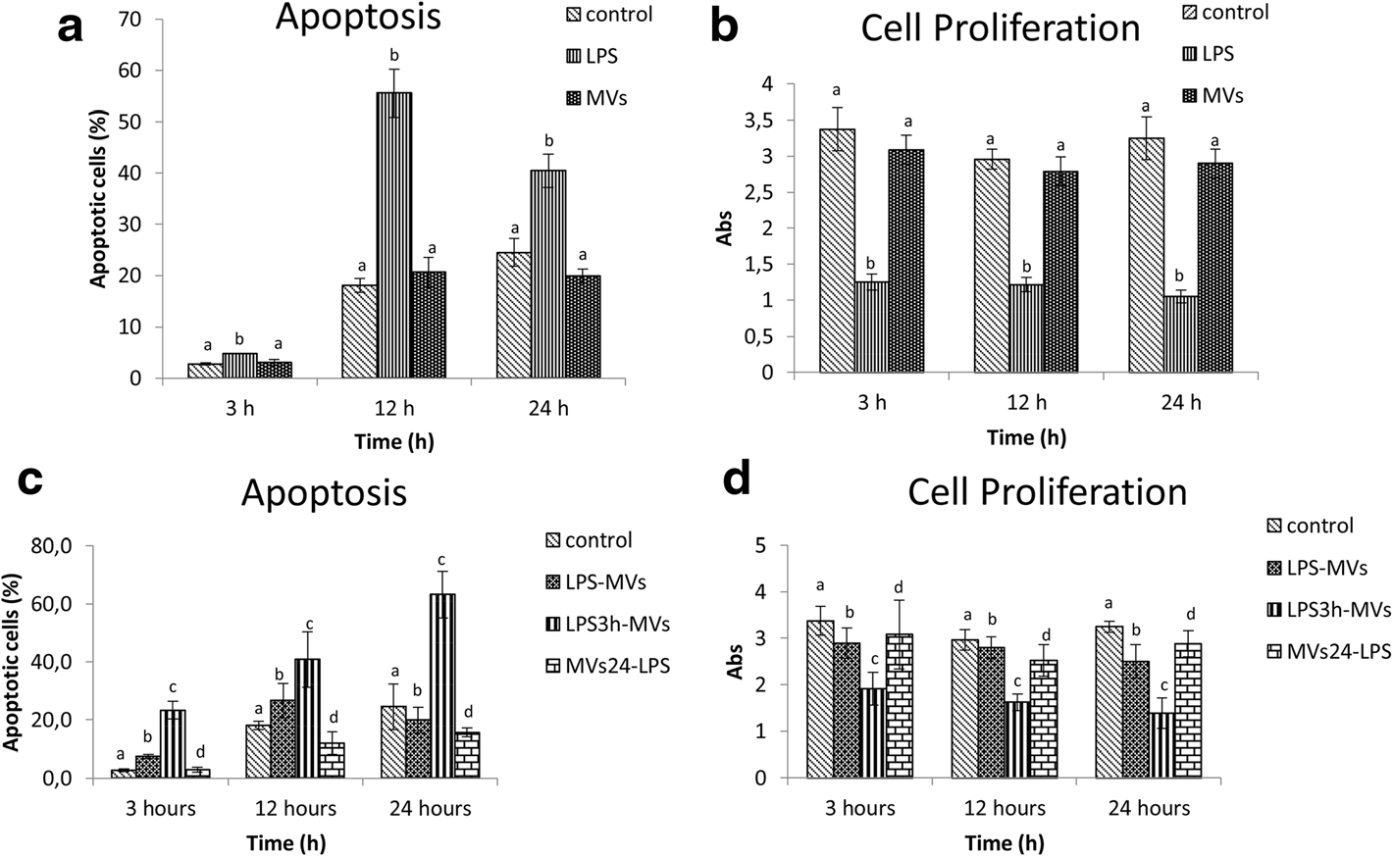
Results of viability and proliferation. The rate of cells at **a** the early stage of apoptosis and **b** absorbance values of the cell proliferation test in control cells. **c** Early apoptosis rate and **d** absorbance values of the cell proliferation test in treated cells. Data represent the mean and SD of three independent experiments. Values labeled with different letters are statistically different within each time point (3 h, 12 h and 24 h) (*P <* 0.05). *MV* microvesicle, *LPS* lipopolysaccharide

The effect of LPS and MVs was evaluated by apoptotic and cell proliferation tests. The rate of cells at the early stage of apoptosis increased dramatically on treatment with LPS; indeed, the percentage of apoptotic cells reached 55 ± 4.1 % at 12 h of stress, decreasing to 40.48 ± 4.82 % at 24 h. The rate of apoptosis due to MVs is not statistically different from endometrial cells alone (Fig. 4a). The results of the cell proliferation test showed the opposite trend to the apoptotic test, confirming the effect of LPS and MVs (Fig. 4b).

MVs were able to counteract the action of LPS either when used simultaneously with LPS or when incorporated from endometrial cells 24 h before the treatment with LPS. In this condition, cells previously treated with MVs and after being exposed to LPS had a lower apoptotic rate (*P* < 0.05) than the control cells, at both 12 and 24 h of experiment (12.01 ± 1.38 % vs 18.05 ± 1.34 % at 12 h and 15.56 ± 1.5 % vs 24.5 ± 2.78 % at 24 h). On the other hand, the stress induced by LPS exposure before the treatment with MVs was not contrasted by MVs and the apoptotic rate increased up to 63.16 ± 6.8 % at 24 h (Fig. 4c). The results of the cell proliferation test showed the opposite trend to the apoptotic test (Fig. 4d).

### Molecular biology study

**Fig. 5:**
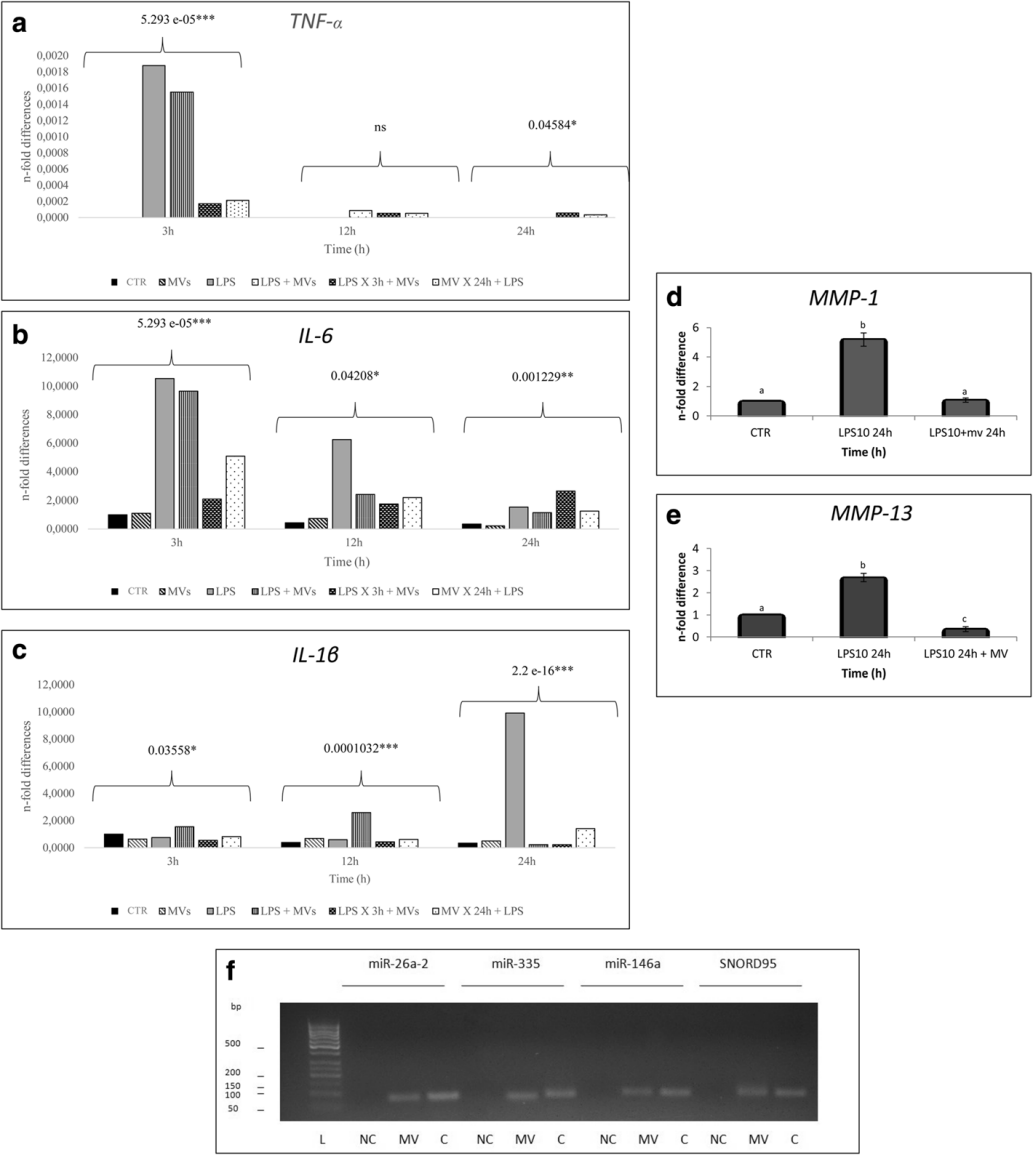
Quantitative RT-PCR analysis for the expression of **a** *TNF-α*, **b** *IL-6*, and **c** *IL-1β* in endometrial cells in different experimental conditions*.* Expression of **d** *MMP-1* and **e** *MMP-13* in endometrial cells exposed to 10 ng/ml LPS (*LPS10 24h*) and simultaneously to 10 ng/ml LPS and MVs (*LPS10 24h + MV*). Expression levels have been normalized to the reference gene (*GAPDH*). Data are represented as fold-change compared with the expression observed in endometrial cells (control, CTR). Values are mean ± SD (*n* = 3). *P* values of expression levels in respect to control are shown above groups when significant in panels **a–c**, and the values labeled with different letters are statistically different (*P <* 0.05) in panels **d** and **e**. **f** PCR assays to determine the presence of different miRNA (miR-26a-2, miR-335, miR146a), and small nucleolar snoRNA, C/D Box 95 (*SNORD95*; positive control) in MVs and cells (*C*) isolated from amniotic membrane. *CTR* control, *IL* interleukin, *L*  molecular weight ladder, *LPS* lipopolysaccharide, *MMP* matrix metalloproteinase, *MV* microvesicle, *NC* negative control, *ns* not significant, *TNF* tumor necrosis factor

The expression of some pro-inflammatory genes was evaluated by qRT-PCR. Endometrial cells, LPS, and MVS were tested alone. Data were obtained from three samples and are shown in Fig. 5. LPS at 3 h significantly upregulated (*P* < 0.05) the expression of *TNF-α* and *IL-6* (0.0019 ± 0.317^E–6^ and 10.54 ± 0.014, respectively) and of *ILβ-1* at 24 h (9.91 ± 0.017). Endometrial cells used as controls (CTR) and MVs did not induce expression of pro-inflammatory genes. In the experiment with simultaneous use of LPS and MVs, the action of LPS was counteracted by MVs; indeed, the expression of *IL-6* at first increased significantly (*P* < 0.05) at 3 h by LPS and then fell significantly in the presence of MVs either at 12 h (2.41 ± 0.039) or at 24 h (1.15 ± 0.081). *IL-1β* at 24 h was completely and significantly (*P* < 0.05) downregulated (0.22 ± 0.0008). The expression of *TNF-α* is not dependent on the presence of MVs.

When LPS was used for 3 h before adding the MVs, the action of LPS was neutralized by the presence of MVs. Indeed, the expression of *IL-6* at 3 h, 12 h, and 24 h was 2.08 ± 0.0019, 1.75 ± 0.0033, and 2.65 ± 0.0013, respectively, compared to the treatment with LPS only. In addition, the expression of *IL-1β* was significantly (*P* < 0.05) downregulated at 24 h (0.21 ± 0.0016).

Under the final condition of MVs for 24 h and then LPS, the expression of all genes was downregulated. *TNF-α* expression at 3 h fell 0.0002-fold compared to the treatment with LPS. *IL-6* expression was statistically (*P* < 0.05) downregulated at each time point, and *IL-1β* was statistically (*P* < 0.05) downregulated at 24 h.

A moderate but significant (*P* < 0.05) increase in the expression was observed for *MMP-1* (5.18 ± 0.44) and *MMP-13* (2.69 ± 0.19) compared to untreated cells when endometrial cells were exposed to LPS for 24 h (Fig. 5d and e). The presence of MVs, simultaneously to LPS, significantly counteracted the effect of LPS on the expression of metalloproteinases as shown by the striking reduction in the expression levels for *MMP-1* (1.67 ± 0.14) and *MMP-13* (0.36 ± 0.11).

### miRNA analyses by RNA extraction and PCR amplification

Expression of specific miRNA was determined by PCR assay that showed the presence of miR-26a-2, miR-335, and miR-146a in both MVs and cells isolated from the amniotic membrane (Fig. 5f).

### Cytokines

**Fig. 6:**
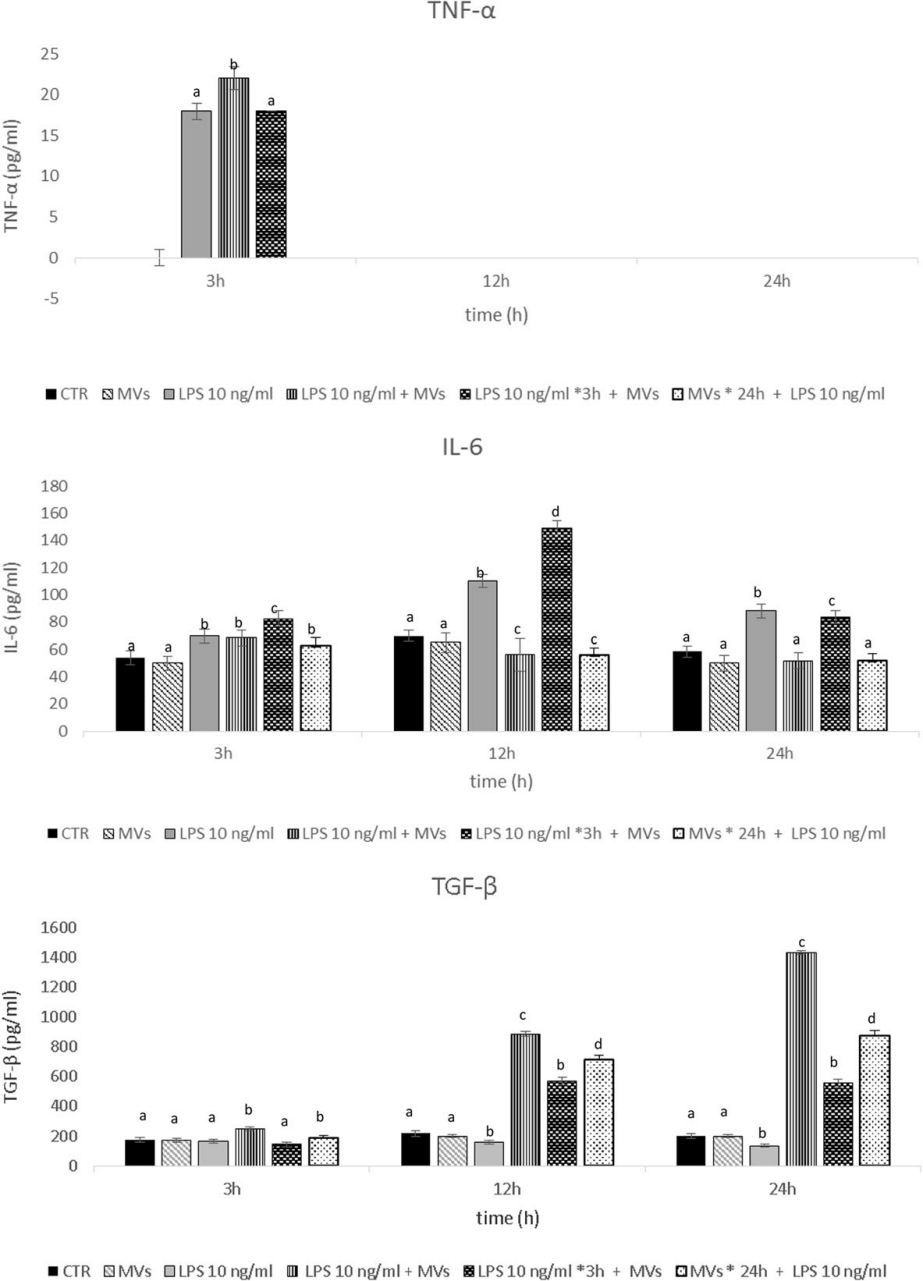
Effect of LPS and MVs under different experimental conditions on the release of TNF-α, IL-6, and TGF-β in endometrial cells. Each value represents the mean ± SD of three samples. Values labeled with different letters are statistically different within each time point (3 h, 12 h and 24 h) (*P <* 0.05) *CTR* control, *IL* interleukin, *LPS* lipopolysaccharide, *MV* microvesicle, *TGF* transforming growth factor, *TNF* tumor necrosis factor

The results of the release over time (3, 12, and 24 h) of pro-inflammatory (TNF-α and IL-6) and anti-inflammatory (TGF-β) cytokines by endometrial cells stimulated with 10 ng/ml of LPS and treated with MVs are shown graphically in Fig. 6.

Cells stressed with LPS secreted significantly (*P* < 0.05) more TNF-α and IL-6 at 3 h and 12 h, respectively, when compared to control cells and cells treated by MVs. The MVs, used simultaneously or incorporated from endometrial cells 24 h before the treatment with LPS, were able to counteract the action of LPS and significantly (*P* < 0.05) and equally decreased the production of TNF-α and IL-6, mainly between 12 h and 24 h. Cells incubated with LPS for 3 h before the treatment with MVs secreted significantly more TNF-α and IL-6 when compared to all the other experimental conditions.

TGF-β was constitutively produced by control cells but the treatment with MVs induced a higher release (*P* < 0.05) of TGF-β, mainly when used simultaneously with LPS or incorporated from endometrial cells 24 h before the treatment with LPS. No efficacy of MVs was evident after LPS-induced stress of cells for 3 h before the addition of MVs.

## Discussion

MVs are secreted by MSCs. Although bone marrow represents the most widely investigated source of MSCs, cells harvested from bone marrow have limited potential in terms of in vitro proliferation capability [43, 44] and do not appear to noticeably improve long-term functionality [45] compared to those from extra-fetal tissues. AMCs have already been demonstrated to be an excellent source for the treatment of tendon diseases in horse [26]. Furthermore, Lange-Consiglio et al. [46] used the conditioned medium derived from AMCs for the treatment of horse tendon diseases and showed that the positive evolution of spontaneous tendon injuries in competition horses was comparable to that achieved with AMCs, thereby showing that AMC-CM had angiogenic and immunomodulatory properties mediated by paracrine mechanisms. Corradetti et al. [39] demonstrated that equine AMCs share the same transcriptional profile as endometrial cells and express genes that are involved in early pregnancy, pre-implantation, and conceptus development. In addition, co-culture of endometrial cells by transwell in the presence of AMCs, or incubated with CM, showed a significant increase in the proliferation rate of endometrial cells compared to fibroblasts and the CM secreted by them. All these preliminary data suggest that AMCs and CM exert regenerative effects through paracrine mechanisms, and that AMCs and their CM may have the potential to improve endometrial cell replenishment. Since MVs are contained in the CM, the aim of the present study was to investigate the role of MVs produced by AMCs in in-vitro cell-to-cell communication that could ultimately lead to endometrium repair. We ultimately aim to demonstrate that they could be used as effective tools for regenerative medicine purposes, especially in the reproduction field.

Our results show that AMCs secrete MVs (with a mean size of 258 nm) as detected by a Nanosight instrument, and this size allows us to categorize them as shedding vesicles. Moreover, we found that MVs are easily internalized by endometrial cells. Fluorescent microscopy analysis suggests that the uptake of MVs by endometrial cells starts at 6 h after co-culture and increases gradually at 24 h, rising to the maximum internalization at 72 h at all different concentrations. At a concentration of 50 × 10^6^ MVs/ml, a decrease at 48 h and 72 h was detected. We hypothesize that after 48 h and 72 h of exposure at 40 × 10^6^/ml, the cells are saturated and phagocytosis of MVs, for the release of their contents into the cell cytoplasm, begins. This phagocytosis probably results in the destruction of the MV membranes and, consequently, in the loss of fluorescence signal. Cocucci et al. [47] demonstrated that the internalization of MVs is the result of the direct fusion or endocytic uptake by target cells. Once internalized, the MVs fuse their membrane with that of endosomes, making a horizontal transfer of their contents into the cytoplasm of the recipient cells. Alternatively, MVs can remain segregated inside the endosomes and be phagocytized by lysosomes or eliminated from the cells after fusion with the plasma membrane through a mechanism of transcytosis [47]. Our data show that horizontal transfer of the contents of MVs within endometrial cells could be one of the mechanisms of action of MVs, although other methods of interaction may occur simultaneously. The process of uptake and internalization of MVs by the endometrial cells could also be facilitated by the presence of surface cell receptors. This hypothesis is confirmed by the results of experiments in which MVs were treated with trypsin before being incubated with endometrial cells. Having identified that the endometrial cells represent target cells for MVs secreted by equine AMCs, a further aim of this study was to understand whether MVs might be involved in the regeneration of endometrial diseases. Obviously, due to the difficulty of studying the repair systems of endometritis in vivo*,* we recapitulated in vitro the inflammatory process by stimulating cells with LPS.

The inflammatory response is a complex process involving many signaling cascades and cytokines have a significant role in the recruitment of inflammatory cells [48]. In the genital tract, the initial response of the endometrium against infection is dependent on innate immunity and mucosal defense systems [49, 50]. The uterine immune response is generated not only by professional immune cells but also by endometrial epithelial and stromal cells, which can respond to LPS through the Toll-like receptors (TLRs) [51]. Activated TLRs subsequently stimulate the production of pro-inflammatory cytokines and chemokines [52].

To understand the mechanism of action of MVs, a single concentration of 40 × 10^6^ MVs/ml was used after stress induced with LPS. This concentration was chosen because, during the study of internalization, endometrial cells at 24 h of culture were not saturated with MVs and no MV degradation started.

In these experiments, we used LPS and MVs under different conditions. In control cells, even if the apoptosis is higher than expected, the cell proliferation of 80 % remaining cells was very high during the 24 h of experimentation, as proven by MTT values. Indeed, viability was constantly high over the different times of culture, and values of absorbance also did not statistically change in the presence of MVs. When the cells were stressed with LPS, the viability decreased with respect to control cells and cells cultured in the presence of MVs. Indeed, apoptosis increased drastically after treatment with LPS and, vice versa, the proliferation declined to the same intensity. The dose of LPS (10 ng/ml) was chosen on the basis of data obtained by Herath et al. [53]. These authors found that this value is present in cows with clinical endometritis. Also, over a short time, this dose of LPS is probably also deleterious for endometrial cells in a static system in vitro if compared to in vivo environments, where this effect can be modulated. These results underscored the stressor effect of LPS that has been reduced by the beneficial effect of MVs on the vitality of cells. When LPS and MVs were used at the same time, the viability of cells was high without significant differences compared to control cells. These results confirm the anti-inflammatory properties of MVs. However, the beneficial effect of MVs is time-dependent. In fact, MVs did not counteract the action of LPS if the cells were previously exposed to LPS (3 h LPS exposure). On the other hand, LPS does not show any detrimental effect if the internalization of MVs in the endometrial cells occurred 24 h before LPS treatment.

The expression of pro-inflammatory genes supports these data. LPS induces overexpression of *IL-1β* at 24 h and MVs are able to counteract this action. Indeed, at 24 h this expression is downregulated in all three experimental conditions (LPS and MVs simultaneously, LPS for 3 h and then MVs, MVs for 24 h and then LPS). This downregulation is observed even in the experiment where cells were previously exposed to LPS. The cells surviving the apoptotic process are probably able to incorporate MVs that, at 24 h, are capable of counteracting the action of LPS. The overexpression of *IL-6* and *TNF-α* induced by LPS occurs at 3 h and, at this time, MVs used either simultaneously or added after the action of LPS are not able to block the action of LPS. This seems to indicate that the downregulation of these genes could occur only when the cells are incubated with MVs for a longer time to guarantee the necessary incorporation of MVs. Indeed, we found that MVs were visible only after 6 h and increased up to 24 h.

In parallel with gene expression, the release of cytokines was studied, confirming the observations regarding gene regulation. LPS was demonstrated to be capable of inducing the release of *TNF-α* the peak of which was obtained 24 h after stimulation with LPS. The release of *IL-6* appeared earlier and reached the highest level 12 h after stimulation with LPS, and then gradually decreased over the subsequent observation period. MVs reduce the release of these pro-inflammatory cytokines and the maximum modulatory activity was observed between 12 h and 24 h, both when MVs were used simultaneously or 24 h before LPS treatment. In both cases, no contrasting action of MVs was obtained within the first 3 h, confirming that the internalization had yet to begin.

*MMP-1* and *MMP-13* expression is induced by *IL-1β*, so the expression of these two genes was investigated at 24 h after treatment with 10 ng/ml LPS, taking into account the higher expression of *IL-1β* at this time. *MMP-1* and *MMP-13* expression was statistically higher compared to the control, confirming the inflammatory effect induced by LPS. Matrix metalloproteinases (MMPs) are a family of structurally related zinc/calcium-dependent proteinases with a pivotal role in the extracellular matrix degradation during both normal and pathological tissue remodeling processes [54, 55]. In addition, the MMP collagenases are key participants in extracellular matrix remodeling and are important for the separation of bovine placental tissues from the endometrium at term [56]. *MMP*-1, -2, -3, -9, and -13 are all highly expressed in the bovine endometrium in late gestation [57], while *MMP-1* and *MMP-13* expression levels are downregulated in postpartum endometrium. Our study demonstrated that MVs downregulated the expression of *MMP-1* and *MMP-13* at 24 h of LPS treatment.

It is well known that miRNA-containing microvesicles can regulate the inflammation process [58]. In this context, from our results it is possible to assume that the cargo of MVs contributed to the anti-inflammatory effect. Since MVs contain various active molecules, such as lipids, proteins, mRNA, and miRNA [29], we studied the presence of three miRNAs in MVs involved in the regulation of pro-inflammatory genes in our in vitro model (miR-335, miR-146a, and miR-26a-2). miR-335 has been demonstrated to regulate the expression of *TNF-a* and *IL-6* during human adipose cell inflammation [59]. miR-146a has been demonstrated to decrease the expression of *IL-1β* and, as an indirect effect, to suppress the level of *MMP* in intervertebral discs in bovine species [60]. miR-26a-2 has been widely studied and it has been correlated with human inflammation, cell proliferation, and apoptosis [61]. The DIANA tool confirmed that these miRNAs have predicted targets also in horse inflammation. The downregulation of gene expression shown in this study could be correlated to miRNA transfer from MVs to endometrial cells.

## Conclusion

These data provide a critical starting point for beginning to dissect how equine amniotic MVs respond to and alter an inflammatory situation, shown to be a promising approach for the treatment of endometritis. Much research is still needed to establish the true biological role of miRNAs in endometrial disease with a view to translating this knowledge into clinically effective outcomes.

## Abbreviations

a.u.: Arbitrary units
AMC: Amniotic mesenchymal stem cell
AOI: Area of interest
CM: Conditioned medium
EDC: Endometrial cell
EGF: Epidermal growth factor
EX: Exosome
FBS: Fetal bovine serum
GAPDH: Glyceraldehyde-3-phosphate dehydrogenase gene
HEPES: N-2-hydroxyethylpiperazine-N-2-ethanesulfonic acid
HG-DMEM: High-glucose Dulbecco’s modified Eagle’s medium
Hoxa9: Homeobox protein Hox-A9-like
IL: Interleukin
LPS: Lipopolysaccharide
miRNA: MicroRNA
MMP: Matrix metalloproteinase
MPR: Membrane-associated progesterone receptor
MSC: Mesenchymal stem cell
MTT: 3-(4,5-dimethylthiazol- 2-yl)-2,5-diphenyletrazolium bromide
MV: Microvesicle
P: Passage
PBS: Phosphate-buffered saline
PGRMC1: Membrane-associated progesterone receptor
PR: Progesterone Receptor
qRT-PCR: Quantitative reverse transcription polymerase chain reaction
RT-PCR: Reverse transcription polymerase chain reaction
SV: Shedding vesicle
TEM: Transmission electron microscopy
TGF: Transforming growth factor
TLR: Toll-like receptor
TNF: Tumor necrosis factor
